# Trends in the Use of Nephron-Sparing Surgery over 7 Years: An Analysis Using the R.E.N.A.L. Nephrometry Scoring System

**DOI:** 10.1371/journal.pone.0141709

**Published:** 2015-11-24

**Authors:** Seung Jea Shin, Kwang Jin Ko, Tae Sun Kim, Hyun Soo Ryoo, Hyun Hwan Sung, Hwang Gyun Jeon, Byong Chang Jeong, Seong Il Seo, Hyun Moo Lee, Han Yong Choi, Seong Soo Jeon

**Affiliations:** Department of Urology, Samsung Medical Center, Sungkyunkwan University School of Medicine, Seoul, Korea; Sun Yat-sen University, CHINA

## Abstract

**Objective:**

To analyze trends in the use of partial nephrectomy, we evaluated which individual factors of renal nephrometry score (RNS) influenced the operative approach bi-annually from 2008 to 2014.

**Materials and Methods:**

We performed a retrospective review of renal cell carcinoma treated by surgery in 2008, 2010, 2012, and 2014. The complexity of renal masses was measured using the R.E.N.A.L. nephrometry scoring system with CT or MRI. Group comparison in terms of operation year and surgical type (partial nephrectomy versus radical nephrectomy) was performed. We developed a nomogram to quantitate the likelihood of selecting partial nephrectomy over radical nephrectomy.

**Results:**

A total of 1106 cases (237 in 2008, 225 in 2010, 292 in 2012, and 352 in 2014) were available for the study. Over the study period, the proportion of partial nephrectomies performed increased steadily from 21.5% in 2008 to 66.5% in 2014 (p < 0.05). Furthermore, use of partial nephrectomy increased steadily in all RNS complexity groups (low, moderate, and high) (p < 0.05). In the analysis of individual components of RNS, values of the R and N components increased statistically by year in the partial nephrectomy group (p < 0.05). Average AUC was 0.920.

**Conclusions:**

The proportion of partial nephrectomies performed sharply increased over the study period. Additionally, over the study period, more partial nephrectomies were performed for renal masses of larger size and closer to the collecting system and main renal vessels. A nomogram developed based on this recent data set provides significant predictive value for surgical decision making.

## Introduction

The prevalence of kidney cancer is on the rise in Korea, as well as in Europe and in the United States. A report from the Korea National Cancer Information Center identified 3,435 new cases in 2009 and 3,989 in 2011 [1].

Open radical nephrectomy has been the treatment of choice for small cortical renal masses for the last 40 years [2]. However, there has been a significant expansion of nephron-sparing surgery in several centers over the past two decades, reflecting a paradigm shift towards preferential usage of partial nephrectomy for the treatment of RCC, especially for small renal tumors [3–5]. Nephron-sparing surgery was initially developed for patients who were not eligible for radical nephrectomy. In this context, imperative indications for partial nephrectomy included anatomical or functional single kidney, chronic kidney disease, or bilateral renal tumors. Long-term studies have now demonstrated that partial nephrectomy has equivalent oncological outcomes compared to radical nephrectomy and has additional benefits including preserving renal function, preventing postoperative chronic kidney disease, providing improved cardiovascular function, and decreasing overall mortality, as discussed in many studies [6, 7]. Several groups have reported that T1a (less than 4 cm) and even T1b (4 to 7 cm) renal cortical tumors can be treated with partial nephrectomy [8–10].

Several factors—including size of the renal mass, exophycity, distance from the collecting system, and location from the hilar structure—determine the suitability of partial nephrectomy for localized cortical tumors. The R.E.N.A.L. nephrometry score (RNS) was developed to quantify the anatomic features of solid renal masses in an objective and reproducible manner [11]. RNS has been shown to correlate with several surgical factors and to predict optimal surgery type [12, 13]. Prior studies have investigated and confirmed its ability to predict histologic type, renal functional outcomes, and pathology [13, 14]. To the best of our knowledge, however, there are limited data evaluating the ability of RNS to predict surgical decision-making (i.e., radical nephrectomy vs. partial nephrectomy). In this context, we reviewed the data for nephrectomy from our institution and analyzed trends in the use of nephron-sparing surgery over a 7-year study period. We assessed how renal mass complexity influenced surgical choices by applying the RNS system to our patient data base and evaluated how individual RNS components contributed to the operative approach chosen by year. In addition, based on a recent data set (RCC data from 2014), we developed a nomogram to quantitate the likelihood of selection of radical nephrectomy vs. partial nephrectomy.

## Methods

### Ethics Statement

This retrospective study was approved by the Samsung Medical Center (SMC) institutional review board. IRB file number is SMC 2015-03-090. Patients’ records and informations were anonymized and de-identified prior to analysis.

### Patients

We performed a retrospective review of 1,217 patients who underwent radical nephrectomy or partial nephrectomy for renal cell carcinoma in 2008, 2010, 2012, and 2014. We excluded 36 patients who had bilateral tumors or had functional or anatomical solitary kidney. We also excluded 57 patients who intended to undergo cytoreductive nephrectomy, 7 patients who were on hemodialysis at the time of surgery, and 10 patients with two or more renal tumors on the ipsilateral kidney. One patient underwent emergency radical nephrectomy for ruptured RCC and was excluded from the study for this reason. Of the remaining 1,106 patients, 568 radical nephrectomy and 538 partial nephrectomy cases were available for this study. All surgical procedures were performed at a single institution and by one of eight surgeons. Based on their surgical experience level, surgeons were classified in three groups: most experience group (two surgeons), moderate experience group (three surgeons), and least experience group (three surgeons). The mean periods from the acquisition of the specialty of urology to study performance were 29 years, 16.4 years, and eight years, respectively. Surgical procedures were performed as one of four types: open surgery, hand-assisted laparoscopic surgery (HALS), laparoscopic surgery, and robotic maneuver surgery. Robotic surgery was performed only for partial nephrectomy cases based on the consensus of our institution. The characteristics we assessed included age, gender, BMI, ASA score, baseline creatinine values, pathologic T stage, and RNS.

### Measurement of R.E.N.A.L. nephrometry scores

A single reviewer calculated the RNS according to criteria developed by Kutikov and Uzzo. In this system, the RNS is composed of renal mass diameter, endophyticity or exophyticity, proximity to the collecting system, location relative to the polar line and hilar structure, and anteriority or posteriority of the mass location [11]. The first four of these features are scored on a 3-point scale. The fifth feature, whether it lies anteriorly or posteriorly to the coronal plane of the kidney, is presented as a descriptor. Based on RNS, lesions are classified as being of low (4–6), moderate (7–9) or high (10–12) complexity.

### Statistical analyses

For comparisons of group by year and surgical types, we used ANOVA and t-tests for continuous variables and chi-square test or Fisher’s exact test for categorical variables (p-value = 0.05). To construct a nomogram for surgical type, affecting factors were selected by stepwise variable selection in logistic regression (p-value = 0.05).

The discrimination power of the nomograms, which predict surgical approach (radical nephrectomy vs. partial nephrectomy), was evaluated by area under the curve (AUC) estimates with 50% indicating no predictive value and 100% indicating perfect prediction. To perform internal validation of our nomograms’ predictive value, we randomly split the sample data set using 5-fold cross-validation and then performed simulation. The nomogram was fit in the training set model.

Statistical analyses were conducted using SAS version 9.4 (SAS Institute, Cary, NC) and R 2.10.0 (Vienna, Austria; http://www.R-project.org). P-values < 0.05 were considered statistically significant.

## Results

Table 1 describes the demographics, perioperative variables, and RNS scores of the study period by year. There were 237 cases in 2008, 225 in 2010, 292 in 2012, and 352 in 2014. There were no significant differences with respect to age, sex, BMI, DM, or preoperative creatinine values by year. The mean renal score decreased from 8.49 in 2008 to 7.95 in 2014 (p < 0.01).

**Table 1:** Clinical and radiographic data for 1106 patients treated surgically for RCC.

Variable	2008 (n = 237)	2010 (n = 225)	2012 (n = 292)	2014 (n = 352)	*P*
**Mean age ± SD, yr**	54.8 ± 11.6	54.6 ± 11.5	54.15± 11.2	56.1 ± 12.5	0.204
**Sex, n (%)**					0.915
**Male**	168 (70.9%)	157 (69.8%)	212 (72.6%)	250 (71.0%)	
**Mean BMI ± SD, kg/m** ^**2**^	25.0 ± 3.5	24.6 ± 3.0	24.8 ± 3.1	25.2 ± 3.1	0.195
**Diabetes, n (%)**	31 (13.1%)	31 (13.8%)	38 (13.0%)	31 (8.8%)	0.200
**Mean of Preoperative Cr± SD, mg/dl**	0.93±0.23	0.90±0.26	0.94±0.26	0.93±0.24	0.323
**ASA, n (%)**					<0.0001
**1**	96(40.5%)	86(38.2%)	104 (35.6%)	105 (29.8%)	
**2**	128(54.0%)	132(58.7%)	169(57.9%)	242(68.8%)	
**3**	13 (5.49%)	7 (3.11%)	19 (6.51%)	5 (1.42%)	
**Mean tumor size, cm**	4.75	4.55	4.04	4.06	0.01
**Pathologic T stage, n (%)**					0.675
**T1**	185(78.1%)	175 (77.8%)	231 (79.1%)	286 (81.2%)	
**T2**	21 (8.9%)	15 (6.7%)	18 (6.2%)	19 (5.4%)	
**T3**	29 (12.2%)	34 (15.1%)	42 (14.4%)	47 (13.4%)	
**T4**	2 (0.8%)	1 (0.4%)	1 (0.3%)	0 (0%)	
**RNS complexity group, n (%)**					<0.0001
**Low complexity**	42(17.7%)	72(32.0%)	102 (34.9%)	102 (29.0%)	
**Moderate complexity**	104(43.9%)	84 (37.3%)	113(38.7%)	158(44.9%)	
**High complexity**	91 (38.4%)	69 (30.7%)	77 (26.4%)	92 (26.1%)	
**Mean RENAL score ± SD**	8.49 ± 2.01	7.95 ± 2.21	7.68 ± 2.19	7.95 ± 2.14	<0.0001
**RNS individual component**					
**R) Tumor size, cm**					0.002
**≤ 4 cm**	111 (46.8%)	126 (56.0%)	185 (63.4%)	212 (60.2%)	
**> 4 but < 7 cm**	78 (32.9%)	53 (23.6%)	70 (24.0%)	89 (25.3%)	
**≥ 7 cm**	48 (20.3%)	46 (20.4%)	37 (12.7%)	51 (14.5%)	
**E) Exophytic/endophytic**					0.130
**≥ 50% exophytic**	87 (36.7%)	99 (44.0%)	116 (39.7%)	134 (38.1%)	
**< 50% exophytic**	108 (45.6%)	83 (36.9%)	138 (47.3%)	167 (47.4%)	
**Entirely endophytic**	42 (17.7%)	43 (19.1%)	38 (13.0%)	51 (14.5%)	
**N) Nearness to sinus**					0.1307
**≥ 7 mm**	48 (20.3%)	50 (22.2%)	70 (24.0%)	86 (24.4%)	
**> 4 but < 7mm**	25 (10.5%)	35 (15.6%)	54 (18.5%)	54 (15.3%)	
**≤ 4 mm**	164 (69.2%)	140 (62.2%)	168 (57.5%)	212 (60.2%)	
**H) (+)**	22 (9.3%)	39 (17.3%)	36(12.3%)	87(24.7%)	<.0001
**L) Location**					0.0822
**Does not cross the polar line**	63 (26.6%)	70 (31.1%)	103(35.3%)	120(34.1%)	
**Lesion crosses the polar line**	39 (16.5%)	51 (22.7%)	60 (20.5%)	66 (18.8%)	
**>50% is across the polar line**	135 (57.0%)	104(46.2%)	129(44.2%)	166 (47.2%)	

Of the individual components of RNS, the proportions of the R component (p = 0.0055), N component (p = 0.002), and H component (p = 0.0008) showed significant differences by year, while the E and L components showed no significant differences by year. The proportion of R scores over 2 increased from 11.76% in 2008 to 19.8% in 2014. The proportion of N scores of 1 decreased from 64.71% in 2008 to 34.62% in 2014, while the proportion of N scores of 3 increased from 19.61% in 2008 to 43.16% in 2014. Additionally, the H (+) proportion among partial nephrectomy cases increased by year (Table 2).

**Table 2:** Yearly proportional change of individual RNS components in the partial nephrectomy group.

Variable	2008 (n = 51)	2010 (n = 86)	2012 (n = 167)	2014 (n = 234)	p
**R) Tumor size, cm**					0.0055
** ≤ 4 cm**	45 (88.24%)	83 (96.51%)	149 (89.22%)	190 (81.2%)	
** > 4 but < 7 cm**	6 (11.76%)	3 (3.49%)	18(10.78%)	41 (17.52%)	
** ≥ 7 cm**	0 (0%)	0 (0%)	0 (0%)	3 (1.28%)	
**E) Exophytic/endophytic**					0.4219
** ≥ 50% exophytic**	25 (49.02%)	40 (46.51%)	82 (49.1%)	95 (40.6%)	
** < 50% exophytic**	23 (45.1%)	36 (41.86%)	72 (43.11%)	108 (41.15%)	
** Entirely endophytic**	3 (5.88%)	10 (11.63%)	13 (7.78%)	31 (13.26%)	
**N) Nearness to sinus**					0.002
** ≥ 7 mm**	33 (64.71%)	41 (47.67%)	66 (39.52%)	81 (34.62%)	
** > 4 but < 7mm**	8 (15.69%)	21 (24.42%)	44 (26.35%)	52 (22.22%)	
** ≤ 4 mm**	10 (19.61%)	24 (27.91%)	57 (34.13%)	101 (43.16%)	
**H) (+)**	0 (0%)	0 (0%)	2(1.2%)	17(7.26%)	0.0008
**L) Location**					0.9794
** Does not cross the polar line**	25 (49.02%)	40 (46.51%)	81(48.5%)	106(45.3%)	
** Lesion crosses the polar line**	9 (17.65%)	20 (23.26%)	37 (22.16)	52 (22.22%)	
** >50% is across the polar line**	17 (33.33%)	26(30.23%)	49(29.34%)	76 (32.48%)	

In comparing radical nephrectomy and partial nephrectomy cases, we found significant differences in age, BMI, ASA scores, and especially RNS scores. The mean total RNS scores were 9.19 in the radical nephrectomy group and 6.63 in partial nephrectomy group (p < 0.0001) (Table 3).

**Table 3:** Comparison of patients managed by radical nephrectomy and partial radical nephrectomy.

Variable	RNx (n = 568)	PNx (n = 538)	p
**Mean age ± SD, yr**	56.4±11.8	53.5±11.6	<.0001
**Sex, n (%)**			0.2231
**Male**	395 (65.9%)	392 (72.9%)	
**Mean BMI ± SD, kg/m** ^**2**^	25.0 ± 3.5	24.6 ± 3.0	0.0096
**Diabetes, n (%)**	68 (12.0%)	63 (11.7%)	0.8929
**Mean preoperative Cr ± SD, mg/dl**	0.94±0.27	0.91±0.23	0.0969
**ASA, n (%)**			0.0315
**1**	96(40.5%)	86(38.2%)	
**2**	128(54.0%)	132(58.7%)	
**3**	13 (5.49%)	7 (3.11%)	
**RNS total score**	9.19	6.63	<.0001
**Individual RNS components**			
**R**	2.02	1.14	<.0001
**E**	1.86	1.66	<.0001
**N**	2.81	1.95	<.0001
**L**	2.46	1.84	<.0001
**H (+), n (%)**	165 (29.0%)	19 (3.5%)	<.0001
**RNS complexity group, n(%)**			<.0001
**Low complexity**	52 (9.2%)	266(49.4%)	
**Moderate complexity**	216(38.0%)	243 (45.2%)	
**High complexity**	300(52.8%)	29(5.4%)	

**Fig 1:**
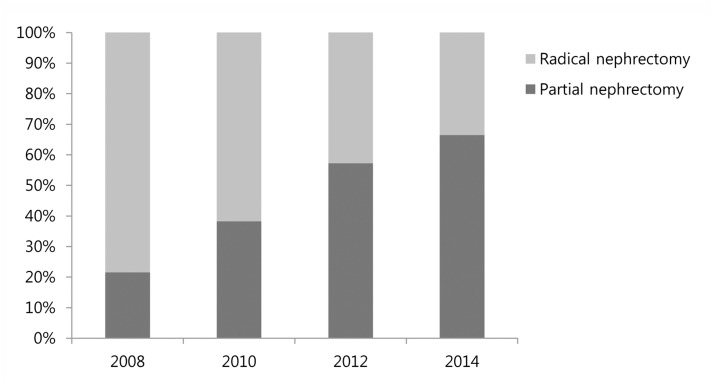
Proportion of patients treated by radical nephrectomy and partial nephrectomy by year.

**Fig 2:**
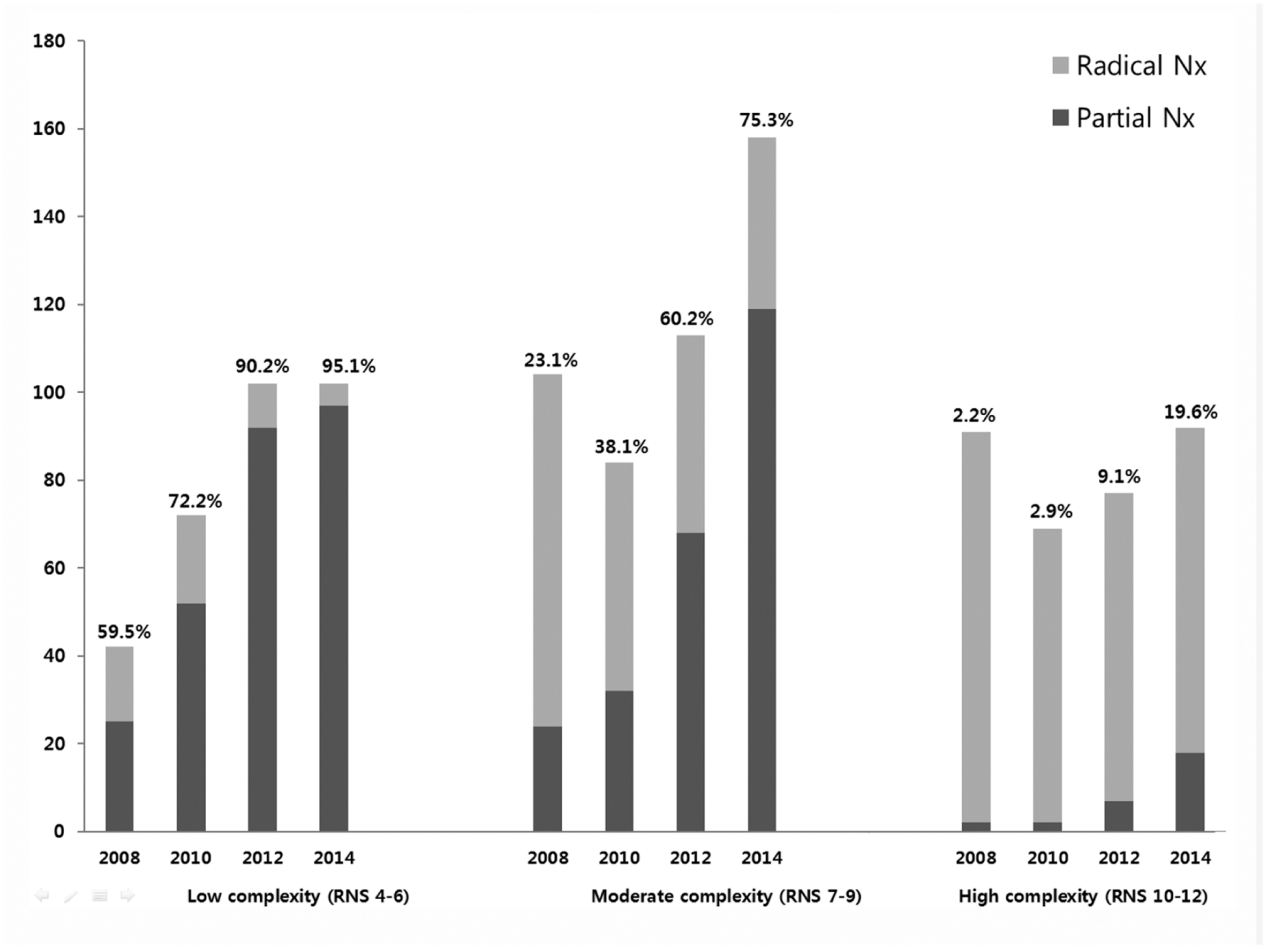
Radical nephrectomy and partial nephrectomy in each RNS group by year. *The percentage of partial nephrectomy is indicated above each bar.

Over the study period, the usage of partial nephrectomy increased steadily, accounting for 21.5%, 38.2%, 57.2%, and 66.5% of all cases, respectively (Fig 1). Additionally, use of partial nephrectomy increased steadily by year in all RNS subgroups (p < 0.05). The proportion of partial nephrectomies increased from 59.5% in 2008 to 95.1% in 2014 in the low complexity group, 23.1% in 2008 to 75.3% in 2014 in the moderate complexity group, and 2.2% in 2008 to 19.6% in 2014 in the high complexity group (Fig 2).

**Fig 3:**
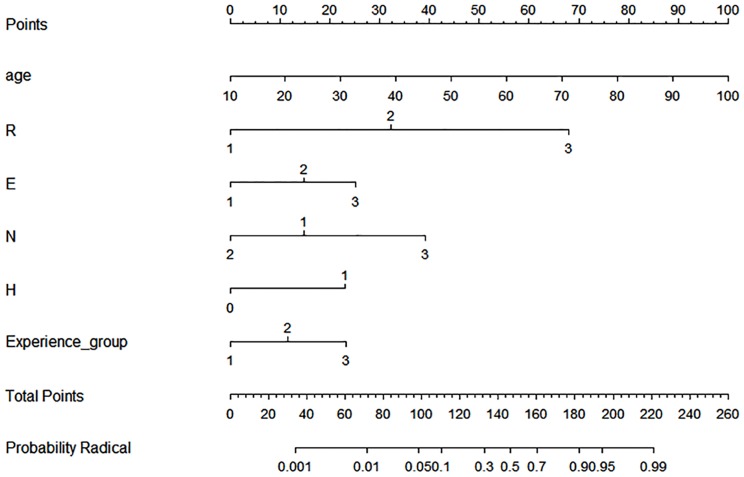
Nomogram (radical nephrectomy vs. partial nephrectomy).

We developed a nomogram based on an RCC data set from 2014 to predict the likelihood that a given renal mass would be treated with radical nephrectomy. Table 4 provides the factors selected in logistic regression. Among the various factors, including basic characteristics, ASA scores, preoperative creatinine, surgeon experience, surgical approach, and individual RNS components, only age (p < 0.0001), surgeon experience (p = 0.0013), the R component (p < 0.0001), E component (p = 0.0056), N component (p = 0.0141) and H (p = 0.0009) component showed statistical significance. The AUC for the partial nephrectomy versus radical nephrectomy model in 5-fold cross-validation was calculated to have a minimum of 0.8975, maximum of 0.9351, and average of 0.920. For the representative display, we used the total data from 2014 (Fig 3).

**Table 4:** Logistic regression analysis of factors affecting surgical decision making (radical nephrectomy vs. partial nephrectomy).

Variable	OR	95% CI	p-value
**Age**	1.071	1.037–1.105	<0.0001
**Experience group**	2.04	1.163–3.578	0.0013
**R component (2 vs. 1)**	7.285	3.201–16.581	<0.0001
**R component (3 vs. 1)**	64.918	15.257–276.219	<0.0001
**E component (2 vs. 1)**	2.486	1.023–6.043	0.044
**E component (3 vs. 1)**	4.707	1.574–14.069	0.0056
**N component (2 vs. 1)**	0.401	0.051–3.172	0.3869
**N component (3 vs. 1)**	4.44	1.35–14.597	0.0141
**H component (positive vs. negative)**	4.154	1.797–9.601	0.0009

## Discussion

Generally, the proportion of partial nephrectomies performed increased sharply over the study period, accounting for 21.5% of cases in 2008 and 66.5% in 2014. This result shows that our institution’s practice reflects a well-established trend of greater nephron-sparing surgery usage at centers of excellence [15–17]. Analysis using the Surveillance, Epidemiology and End Results cancer registry (SEER 1999–2006) showed that the ratio of partial nephrectomy to radical nephrectomy has increased annually, representing 45% for small renal tumors (≤4 cm) in 2006 [15]. Analysis of combined data from six tertiary care centers in Europe demonstrated that partial nephrectomy represented 50% of all nephrectomy cases from 2004 to 2007 [17].

In the same period, the mean tumor size decreased over time from 4.75 cm in 2008 to 4.06 cm in 2014. Likewise, RNS showed a clear decreasing tendency from 8.49 in 2008 to 7.95 in 2014 (p-value < 0.001) (Table 1). This may reflect increasing early detection of renal mass lesions, or a shift toward surgical treatment of lesions which in the past would have been under the threshold of detection. We suspect that this is largely due to widespread routine health check-ups including abdominal ultrasonography, CT, and MRI [18]. In analysis of the partial nephrectomy group, however, we noticed an increasing pattern in RNS over time; we identified increase in RNS from 6.49 in 2008 to 6.89 in 2014 (P-value = 0.01). This increase might infer that surgeons with more experience are more likely to choose partial nephrectomy in complex cases. Likewise, in our study data of 2014, the proportion of partial nephrectomies was 78.9% in the most experience group, 67.1% in the moderate experience group, and 50.8% in the least experience group (p-value = 0.01).

In this study, we performed comparison analysis on the usage of partial nephrectomy based on RNS. Satasivam et al. [3] reported that the increase in the proportion of partial nephrectomies was principally in cases of low complexity, representing 22.2% in 2005 compared with 70.6% in 2009. In their study, there was no significant increase in partial nephrectomy usage in the moderate complexity group over time. In contrast, the increase in partial nephrectomy usage at our institution represents an increase in surgery performed for lesions of all complexities. The usage of partial nephrectomy was 95.1% in 2014 compared with 59.5% in 2008 for low complexity groups. Notably, the frequency of partial nephrectomy for moderate complexity cases was 75.3% in 2014 compared with 23.1% in 2008. For high complexity cases, the frequency was 19.6% in 2014 and 2.2% in 2008.

In this study, we confirmed the increasing trend of partial nephrectomy and evaluated which components, including individual components of RNS, contributed to partial nephrectomy over the study period. In 2012, all patient groups with R score 3 underwent radical nephrectomy, whereas in 2014, three patients with R score 3 were treated with partial nephrectomy. In general, the point values of the R and N components significantly increased by year in the partial nephrectomy group. The H (+) proportion among partial nephrectomy cases also steadily increased during this time (Table 2). These results suggest that over time, surgeons performed nephron-sparing surgery in renal masses with larger size and closer to the collecting system and main renal vessels.

Recently, renal tumor contact surface area, a parameter for predicting complexity and outcomes of partial nephrectomy, was developed [19]. Unlike RNS, contact surface area combines just two aspects of tumor complexity: mass size and proportion of endophytic component. In this context, we assume that mass size (R component), endophycity (E component), and distance to sinus (N component) may have a greater impact on tumor complexity than the other components. Through the nomogram we developed with stepwise tool, we can infer that the choice between partial nephrectomy versus radical nephrectomy depends on the maximum tumor size, the exophytic/endophytic properties of the tumor, the proximity to the collecting system and main renal artery and vein, and age, but does not depend on the tumor location relative to the polar line (L component) or preoperative renal function. In our study data, the mean age of the partial nephrectomy group (53.5 years) was less than that of the radical nephrectomy group (56.4 years) (p <.0001). Furthermore, age was a contributing factor in the predictive nomogram. Although whether partial nephrectomy is superior to radical nephrectomy in terms of overall survival remains controversial [20], these results may promote performing partial nephrectomy in younger patients. Several studies have confirmed that radical nephrectomy causes significant decreases in postoperative renal function when compared to partial nephrectomy [6, 7]. However, preoperative creatinine, representing renal function, did not influence the type of surgery performed in our study.

We note that our study had several limitations. In this study, all RNS was measured by single urologist. This may cause measurement bias in the scoring system due to its radiologic complexity. Although RNS has been shown to have high reproducibility, as suggested by M. Francesca et al., correlation in RNS between urologists and radiologists in RNS could yield more fidelity [21, 22]. Robotic surgery was performed only in cases of partial nephrectomy because of the consensus of our institution. Another limitation of our study is that analysis was performed biannually, not annually. For more precise analysis on trends, we may need an annual, consecutive study. The retrospective design of current study is another limitation. Also, the current study did not assess the potential for postoperative complications, including urine leakage after partial nephrectomy, renal function decreases or cardiovascular diseases. Ultimately, to assess the usefulness of RNS in surgical decision making, further investigation on postoperative complications is necessary.

## Conclusions

Over the study period, the proportion of partial nephrectomies increased sharply from 21.5% in 2008 to 66.5% in 2014. In our analysis according to RNS, the usage of partial nephrectomy increased steadily by year in all RNS subgroups. Surgeons are using partial nephrectomy in renal masses with larger size and closer to the collecting system and main renal vessels. A nomogram based on a recent data set of RCC from 2014 provides significant predictive value in surgical decision making (radical nephrectomy vs. partial nephrectomy).
